# Anti-Inflammatory Activity of *Cannabis sativa* L. Extract in 2,4-Dinitrochlorobenzene-Induced Dermatitis in Rats

**DOI:** 10.3390/ph18030370

**Published:** 2025-03-05

**Authors:** Renata Wolińska, Maria Zalewska, Piotr Poznański, Agata Nawrocka, Agnieszka Kowalczyk, Mariusz Sacharczuk, Magdalena Bujalska-Zadrożny

**Affiliations:** 1 Department of Pharmacotherapy and Pharmaceutical Care, Faculty of Pharmacy, Medical University of Warsaw, Banacha 1, 02-097 Warsaw, Poland; 2 Department of Experimental Genomics, Institute of Genetics and Animal Biotechnology, Polish Academy of Science, Postępu 36A, 05-552 Jastrzębiec, Poland

**Keywords:** dermatitis, *Cannabis sativa* L., cannabidiol (CBD), rats

## Abstract

**Background:** *Cannabis sativa* L. and its products are becoming popular for the treatment of inflammatory diseases. One of the main phytocannabinoids contained in cannabis is cannabidiol (CBD), which is a component of numerous cosmetic preparations used to treat inflammatory skin diseases such as atopic dermatitis (AD) and psoriasis. However, current data regarding the efficacy and safety of CBD for dermatological indications are limited. Therefore, the aim of the present study was to evaluate the anti-inflammatory effect of high-CBD *Cannabis sativa* L. extract (eCBD) in a model of AD. **Methods:** Dermatitis was induced by repeated application of 2,4-dinitrochlorobenzene (DNCB) to the skin of the rats’ ears. The therapeutic effect of eCBD was evaluated in behavioral, histopathological, and hematological studies following topical application as an ointment containing 2% CBD. **Results:** Application of the ointment containing eCBD resulted in attenuation of DNCB-induced inflammation. Interestingly, an anti-edematous effect was more pronounced in rats treated with the eCBD than in rats treated with 1% hydrocortisone ointment. However, eCBD did not reduce the frequency of DNCB-induced scratching, while there was a visible antipruritic effect of 1% hydrocortisone application. Histopathological analysis revealed that both eCBD and 1% hydrocortisone ointments significantly decreased mast cell count compared with the Vaseline control group. Furthermore, treatment with an ointment containing eCBD resulted in a decrease in the number of leukocytes in the blood. **Conclusions:** Topically administered eCBD had a stronger anti-edematous effect than glucocorticosteroid and differently affected hematological parameters. It is suggested that eCBD has therapeutic potential for the treatment of AD.

## 1. Introduction

Atopic dermatitis (AD) is a chronic inflammatory skin disease that manifests in dry skin, erythematous lesions, and intense pruritus [1]. Patients with AD often also show other symptoms related to impaired barrier function of the epidermis, like bronchial hyperresponsiveness, eosinophilic airway inflammation, and respiratory symptoms. Thus, the disease is often accompanied by allergic rhinitis and/or bronchial asthma and substantially impairs quality of life in AD patients, beginning in infancy and childhood and continuing to adulthood [2]. The etiology of AD includes disorders of immune processes, including dysfunction of immunoglobulin E (IgE) type 1, defects in immune response cells, and changes related to epidermal barrier dysfunction. It is postulated that both genetic and environmental factors (e.g., detergents, contact allergens, exposure to house dust mites) are responsible for the development of AD [3].

Histological characteristics of AD are highly similar to those observed in allergic contact dermatitis and depend on the acuity of the skin lesion. In acute lesions, marked intercellular edema (spongiosis) of the epidermis is observed. Moreover, there is a pronounced perivascular infiltration of lymphocytes, dendritic cells, monocytes, macrophages, and single eosinophils in the dermis. Normal numbers of mast cells are present, although in various degrees of degranulation. In turn, chronic lichenified lesions of AD are characterized by hyperplasia of the epidermis. In this stage of disease, little or no spongiosis is described. In addition, chronic atopic lesions may show hyperkeratosis or dyskeratosis and scale crusts. The number of mast cells is significantly increased, and the cells are fully granulated [4,5,6].

The gold standard of treatment for AD flare-ups is topical corticosteroids due to their pronounced anti-inflammatory, antiproliferative, and immunosuppressive effects. However, side effects such as telangiectasia, rosacea, skin atrophy, and impaired wound healing severely limit their use. As an alternative to topical corticosteroids, calcineurin inhibitors may be considered. Furthermore, due to the fact that histamine is well known as one of the pruritogenic mediators, antihistamines have traditionally been used in adjunctive therapy for AD. However, clinical data on their antipruritic effect in AD are limited; therefore, these drugs may be indicated in patients with comorbidities such as allergic rhinitis, urticaria, and sleep disturbances [7,8].

*Cannabis sativa* L. (Cannabaceae) is one of the plants with the longest history of being known and used by humanity for multiple therapeutical purposes. The main active substances in this plant are phytocannabinoids, among others Δ-9-tetrahydrocannabinol (THC) and cannabidiol (CBD). THC is a high-affinity partial agonist of both CB1 and CB2 cannabinoid receptors. There are few in vitro and in vivo studies concerning THC effects in the context of inflammatory skin diseases, but the central side effects of the compound are a serious burden [9,10,11]. In turn, CBD has a low functional activity on the CB1 receptor, whereas the compound is a weak inverse CB2 agonist [12].

Interestingly, many of CBD’s pharmacological effects (including its anti-inflammatory effect) result not only from CBD direct action on membrane receptors, but also from its modification of endocannabinoid tone. Cannabidiol can alter the uptake of anandamide and thus enhance the anti-inflammatory effect of endocannabinoids. The mechanism of CBD’s anti-inflammatory action is also associated with 5HT1A and PPARγ receptors. Cannabidiol is an agonist of the adenosine A2A receptors and therefore may cause a reduction in the level of the pro-inflammatory cytokine TNF-α. Moreover, the pharmacological activities of CBD include an effect on the fibrotic response and apoptosis processes, as well as modulation of the functions of immune system cells, reducing the level of oxidative stress and affecting ion channels [13,14].

Therefore, non-psychoactive CBD is one of the most valuable cannabinoids because of its complex anti-inflammatory, analgesic, anxiolytic, and antiepileptic effects. Moreover, topical formulations containing CBD are used in the treatment of various dermatological diseases, despite the fact that current data regarding their efficacy and safety are limited [15]. Del Rosso [16] examined the effectiveness of a non-steroidal cream containing, palmitoylethanolamide (PEA), endocannabinoid-like lipid mediator in patients with AD and observed that treatment with a mid-potency topical corticosteroid, clocortolone pivalate 0.1% cream, in combination with the PEA-containing product, resulted in faster skin clearance in comparison to treatment with the corticosteroid with moisturizer cream only. In turn, to our knowledge, there have been few attempts to assess the effects of preparations containing CBD in patients with AD [17]; however, these were observational studies, lacking a control group receiving established treatments, e.g., glucocorticoids. Interestingly, there is a growing body of evidence that the endocannabinoid system plays a significant role in the physiological processes in the skin, as well as in skin diseases. It has been observed that endocannabinoids and cannabinoid receptors are up-regulated in the skin of animals and humans with AD [18,19,20,21,22].

Building on the studies described above, the aim of the present study was to evaluate the anti-inflammatory effect of high-CBD *Cannabis sativa* L. extract (eCBD) in a rat model using 1-chloro-2,4-dinitrobenzene (DNCB)-induced dermatitis. The effect of the ointment containing eCBD was compared with that of the ointment vehicle, Vaseline, and with that of an ointment containing the glucocorticosteroid hydrocortisone.

## 2. Results

### 2.1. Topical Application of Ointment Containing High-Cannabidiol Cannabis sativa L. Extract Resulted in Attenuation of DNCB-Induced Ear Edema in Rats

**Figure 1:**
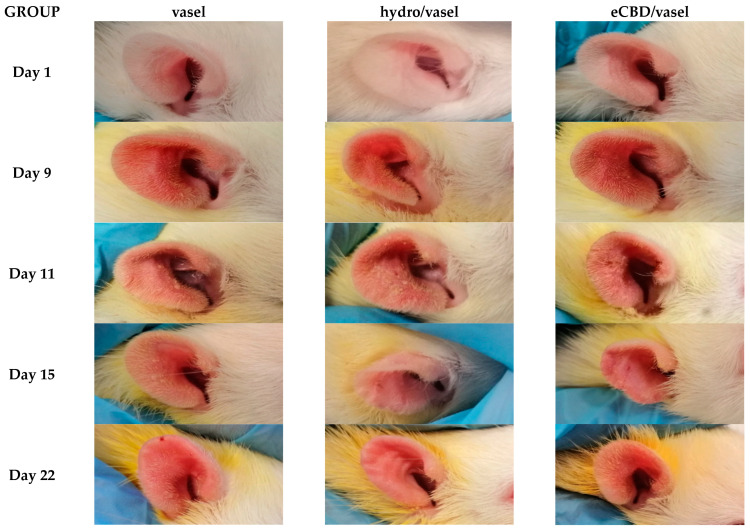
Therapeutic effect of high-cannabidiol *Cannabis sativa* L. extract (eCBD) ointment on DNCB-induced dermatitis in rats. The effect of the ointment containing 2% cannabidiol (eCBD/vasel) was compared to those of Vaseline (vasel) and ointment containing 1% hydrocortisone (hydro/vasel), which served as an active control. The 2,4-dinitrochlorobenzene (DNCB) solution was administered 9 times over a period of 3 weeks, and the tested ointments were applied starting on day 11. The application of the ointments containing eCBD and hydrocortisone resulted in the alleviation of inflammatory lesions compared to Vaseline, as observed on days 15 and 22. Treatment with eCBD resulted in a reduction of redness and swelling compared to day 9, before treatment.

The administration of DNCB to both surfaces of the animals’ right ears resulted in local inflammation, as manifested in ear redness and edema. The developing inflammatory changes were also associated with increased, intense scratching of the ear, which especially intensified immediately after application of the compound. In addition, peeling of the epidermis and the appearance of small wounds and scabs on the animals’ ears were observed (Figure 1).

Inflammation developed gradually, and on the 10th day of the experiment, significant differences were observed between the negative control (70% ethanol) and animals that had received the DNCB solution. On day 10, before the initiation of treatment, ear thickness was 1.26 ± 0.04 mm in the DNCB group, as opposed to 0.67 ± 0.02 mm in the negative control group.

**Figure 2:**
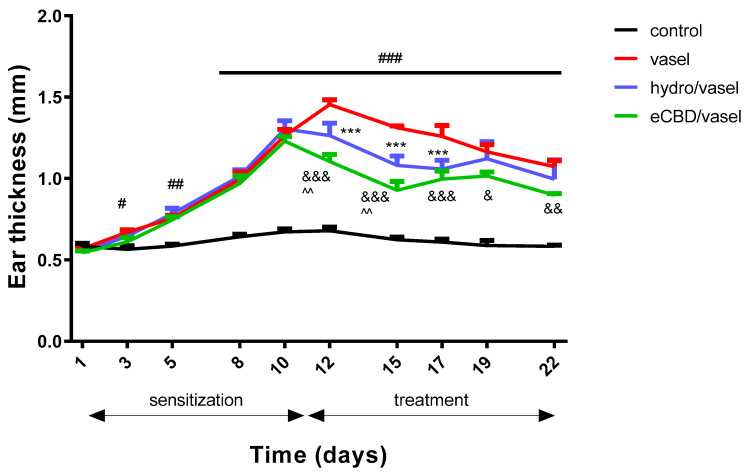
The effect of high-cannabidiol *Cannabis sativa* L. extract (eCBD) ointment on ear edema on DNCB-induced dermatitis. The 2,4-dinitrochlorobenzene (DNCB) solution was administered 9 times over a period of 3 weeks, and the tested ointments containing 2% cannabidiol (eCBD/vasel), hydrocortisone 1% (hydro/vasel), or vehicle–Vaseline (vasel) were applied beginning on from day 11. The severity of edema was assessed by measuring the ear thickness (mm). Treatment with eCBD resulted in the significant reduction of ear thickness, and the effect was more pronounced than anti-edematous effect observed after administration of hydrocortisone 1%. The control group consisted of animals without inflammation. The results are presented as mean ± SEM, n = 7–8. # *p* < 0.05; ## *p* < 0.01; ### *p* < 0.001 control vs. vasel; *** *p* < 0.001 hydro/vasel vs. vasel; & *p* < 0.05, && *p* < 0.01, &&& *p* < 0.001 eCBD/vasel vs. vasel; ^^ *p* < 0.01 hydro/vasel vs. eCBD/vasel.

Treatment with an ointment containing eCBD was started on the 11th day of the experiment. The application of ointment containing eCBD resulted in a significant alleviation of inflammatory lesions compared to Vaseline. Interestingly, the anti-edematous effect occurred after the first ointment application, on the 12th day of the study, and was long-lasting. Similarly, a significant anti-edema effect of the ointment containing 1% hydrocortisone was observed from the 12th day of the study. Moreover, the anti-inflammatory effect of eCBD seems to be more pronounced compared to that of hydrocortisone 1%, as significant differences in the ear thickness were observed between these groups. However, it is worth considering that the compounds were administered in different concentrations. The ear thickness values measured on day 12 were as follows: 0.68 ± 0.02 mm—control group; 1.45 ± 0.03 mm—Vaseline; 1.26 ± 0.08 mm—hydrocortisone; and 1.10 ± 0.04 mm—eCBD (Figure 2).

### 2.2. Topical Application of Ointment Containing High-Cannabidiol Cannabis sativa L. Extract Did Not Reduce the Frequency of Scratching but Slightly Alleviated the Symptoms of Body Shaking Caused by DNCB Application in Rats

During the development of inflammation, significant intensification of scratching occurred from the 8th day of the experiment and in the Vaseline group it persisted until the end of the experiment. Moreover, the application of DNCB to both surfaces of the animals’ right ears resulted in a significant increase in the frequency of body shakes, starting from the 5th day in the Vaseline group.

**Figure 3:**
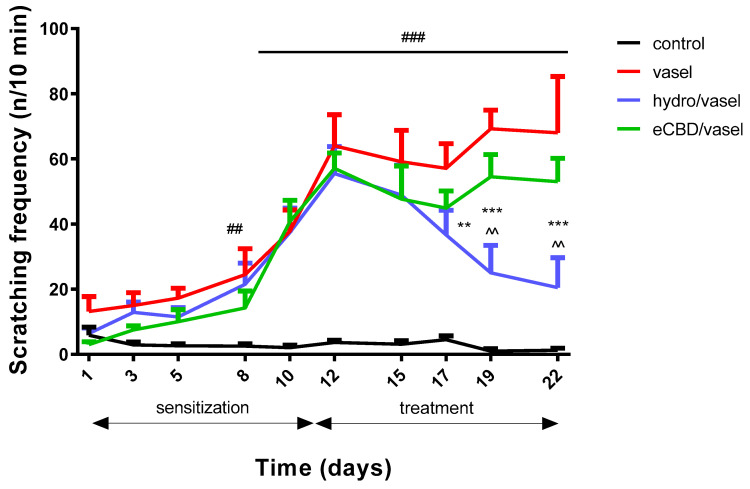
The effect of high-cannabidiol *Cannabis sativa* L. extract (eCBD) ointment on scratching frequency on DNCB-induced dermatitis. The 2,4-dinitrochlorobenzene (DNCB) solution was administered 9 times over a period of 3 weeks, and the tested ointments, which contained 2% cannabidiol (eCBD/vasel), hydrocortisone 1% (hydro/vasel), or vehicle–Vaseline (vasel), respectively, were applied from day 11. Behavioral assessment of the pruritus was performed immediately after DNCB application; the number of scratching events was counted. Treatment with eCBD did not significantly change the scratching frequency compared to treatment with Vaseline. By contrast, the administration of 1% hydrocortisone resulted in a significant reduction in the frequency of scratching. The control group consisted of animals without inflammation. The results are presented as mean ± SEM, n = 7–8. ## *p* < 0.01; ### *p* < 0.001 control vs. vasel; ** *p* < 0.01; *** *p* < 0.001 hydro/vasel vs. vasel; ^^ *p* < 0.01 hydro/vasel vs. eCBD.

In contrast to its anti-edematous effect, administration of eCBD did not affect the degree of itching in tested animals. The frequency of scratching in the group receiving eCBD was not significantly different from that in the group treated with Vaseline and remained relatively constant from day 12. The administration of 1% hydrocortisone resulted in a significant reduction in the intensity of itching, as manifested in a significant decrease in the frequency of scratching from day 17 compared to treatment with Vaseline (Figure 3).

**Figure 4:**
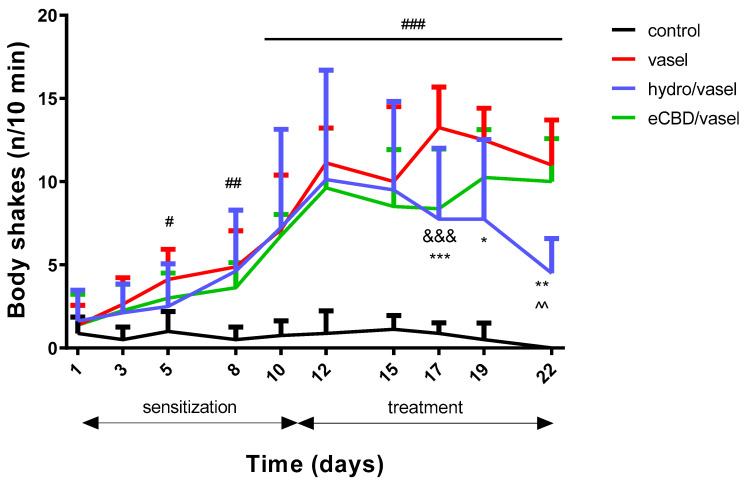
The effect of high-cannabidiol *Cannabis sativa* L. extract (eCBD) ointment on body shakes frequency on DNCB-induced dermatitis. The 2,4-dinitrochlorobenzene (DNCB) solution was administered 9 times over a period of 3 weeks, and the tested ointments, which contained 2% cannabidiol (eCBD/vasel), hydrocortisone 1% (hydro/vasel), or vehicle–Vaseline (vasel), were applied from day 11. Behavioral assessment was performed immediately after DNCB application, and treatment with eCBD resulted in a slight reduction in body shaking compared to treatment with Vaseline. A similar but more pronounced effect was observed in the hydrocortisone 1% group. The control group consisted of animals without inflammation. The results are presented as mean ± SEM, n = 7–8. # *p* < 0.05; ## *p* < 0.01; ### *p* < 0.001 control vs. vasel; * *p* < 0.05; ** *p* < 0.01; *** *p* < 0.001 hydro/vasel vs. vasel; &&& *p* < 0.001 eCBD vs. vasel; ^^ *p* < 0.01 hydro/vasel vs. eCBD.

Interestingly, topical application of ointment with eCBD resulted in a slight alleviation of body shaking compared to application of Vaseline. However, this effect was observed only on day 17 of the experiment. Hydrocortisone caused a significant reduction in body shaking, beginning on 17 day of the study, compared to Vaseline (Figure 4).

### 2.3. Histopathological Evaluation of the Ear Skin Treated with High-Cannabidiol Cannabis sativa L. Extract in a Rat Model of DNCB-Induced Dermatitis

**Figure 5:**
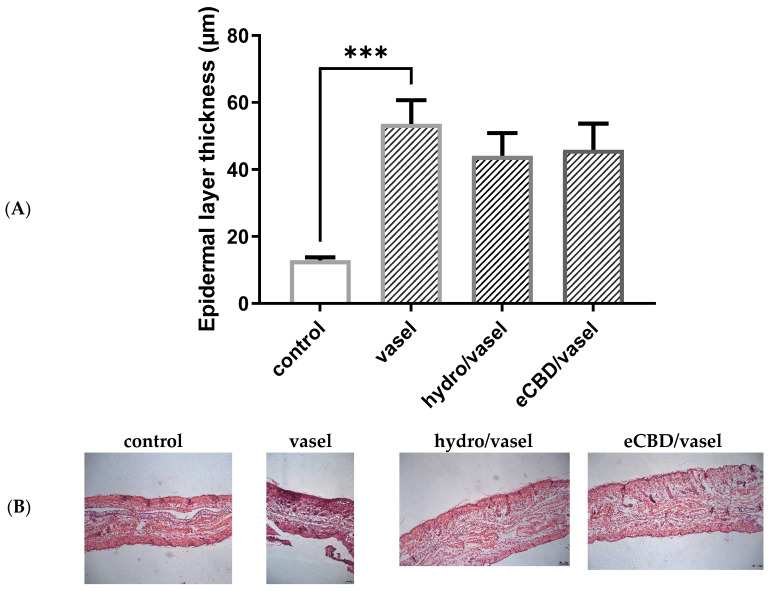
The effect of high-cannabidiol *Cannabis sativa* extract (eCBD) ointment on mean epidermal-layer thickness (**A**), and hematoxylin-eosin (H&E) staining of 5 μm paraffin-embedded sections from the skin of the rat’s ear (**B**) on DNCB-induced dermatitis. The 2,4-dinitrochlorobenzene (DNCB) solution was administered 9 times over a period of 3 weeks, and the tested ointments, which contained 2% cannabidiol (eCBD/vasel), hydrocortisone 1% (hydro/vasel), or vehicle–Vaseline (vasel), respectively, were applied from day 11. The repeated application of DNCB resulted in a significant increase in epidermal-layer thickness in inflamed skin treated with Vaseline. Treatment with either the ointment containing eCBD or the ointment containing hydrocortisone did not change epidermal-layer thickness in those groups compared to the Vaseline group. The control group consisted of animals without inflammation. In the graphs, the bars filled in with gray correspond to groups sensitized with DNCB. The results are presented as mean ± SEM, n = 6–8, *** *p* < 0.001 (**A**). Representative images were photographed under final magnification of 40× (**B**).

**Figure 6:**
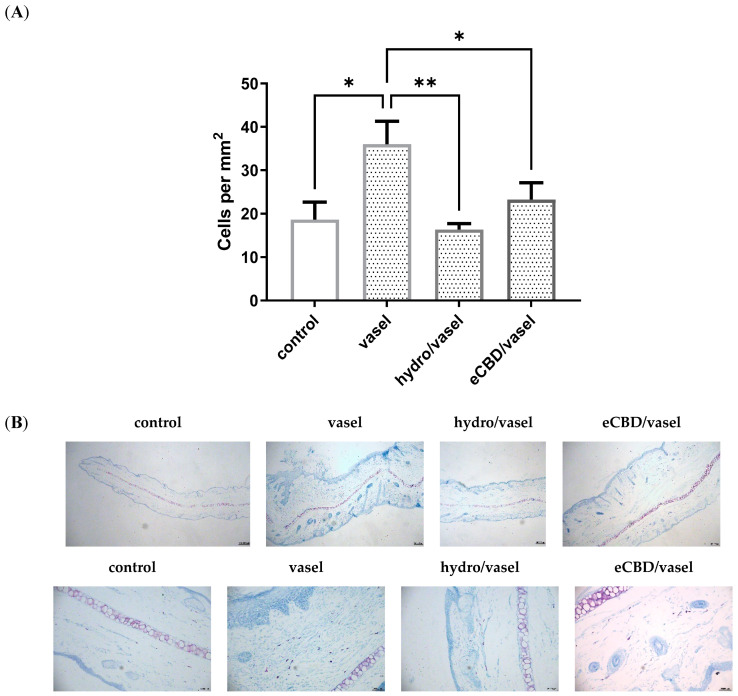
The effect of high-cannabidiol *Cannabis sativa* L. extract (eCBD) ointment on mast cell number in DNCB-induced dermatitis. (**A**) The effect of the topical application of a *Cannabis sativa* L. extract ointment containing 2% cannabidiol (eCBD/vasel) on mast cell number was compared to that of hydrocortisone 1% (hydro/vasel), or vehicle–Vaseline (vasel). The 2,4-dinitrochlorobenzene (DNCB) solution was administered 9 times over a period of 3 weeks, and the tested ointments, which contained 2% cannabidiol (eCBD/vasel), hydrocortisone 1% (hydro/vasel), or vehicle–Vaseline (vasel), respectively, were applied beginning on day 11. Repeated application of DNCB resulted in a significant increase in the mast cell number in inflamed skin, which was treated with Vaseline. Treatment with the ointment containing eCBD, as well as with ointment containing hydrocortisone, decreased mast cell infiltration in the rat skin compared to treatment with Vaseline. The control group consisted of animals without inflammation. In the graphs, the bars filled in with the gray pattern correspond to groups sensitized with DNCB. The results are presented as mean ± SEM (n = 3–4). * *p* < 0.05; ** *p* < 0.01. (**B**) Toluidine blue staining of 5 μm paraffin-embedded sections from the skin of the rat’s ear. Representative images; final magnifications were 40× (upper panel) and 200× (lower panel).

Repeated application of DNCB to the right ear of the rats resulted in a significant increase in epidermal-layer thickness and an increase in the mast cell number in inflamed skin, which was treated with Vaseline from the 11th day of the experiment. Treatment with the ointment containing *Cannabis sativa* L., as well as with the ointment containing hydrocortisone, from day 11 decreased mast cell infiltration in the rat skin compared to treatment with Vaseline. However, neither eCBD nor hydrocortisone treatment changed epidermal-layer thickness compared to treatment with Vaseline (Figure 5 and Figure 6).

### 2.4. Haematological Parameters of Rats Treated with the Ointment Containing High-Cannabidiol Cannabis sativa L. Extract in the Model of DNCB-Induced Dermatitis

#### 2.4.1. Treatment of DNCB-Induced Dermatitis with an Ointment Containing High-Cannabidiol *Cannabis sativa* L. Extract Resulted in a Decrease in the Number of Leukocytes

Induction of dermatitis followed by treatment with Vaseline, ointment with hydrocortisone 1%, or ointment containing eCBD did not result in any changes in total leukocyte count (WBC) compared to the negative control. Interestingly, in rats with dermatitis that were treated with the ointment containing eCBD, a significant decrease in WBC, compared to the groups treated with Vaseline or hydrocortisone, was observed. Moreover, the use of the ointment with eCBD resulted in a reduction in the lymphocyte (LYM) population compared to the negative control and the hydrocortisone group. A slight reduction in the number of monocytes (MONO) was also observed in the eCBD group compared to the other groups; however, statistical significance was not confirmed. Furthermore, there was a trend towards an increase in the granulocyte (GRAN) population in the groups treated with Vaseline or hydrocortisone ointment compared to the negative control.

**Figure 7:**
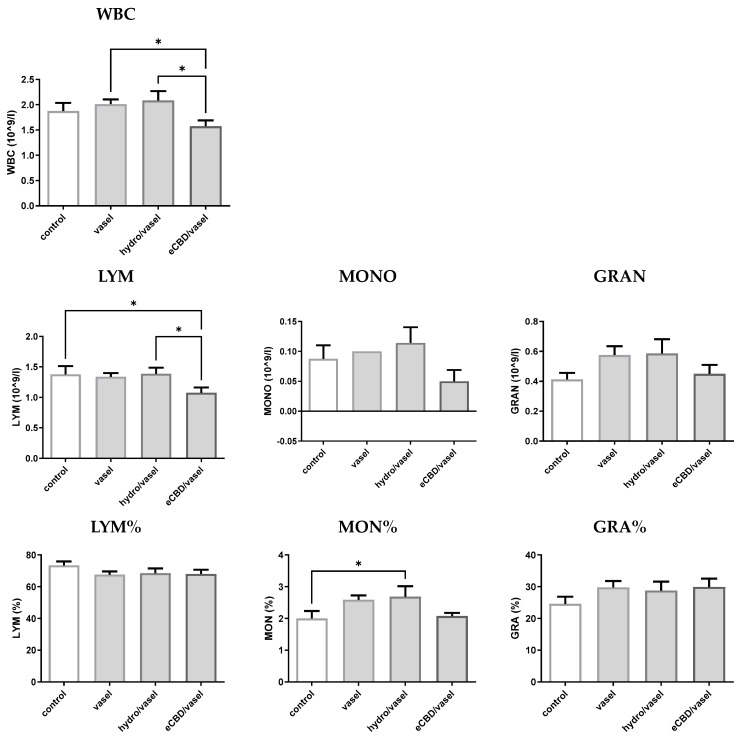
The effect of high-cannabidiol *Cannabis sativa* extract (eCBD) ointment on white blood cell parameters in DNCB-induced dermatitis. The 2,4-dinitrochlorobenzene (DNCB) solution was administered 9 times over a period of 3 weeks, and the tested ointments, which contained 2% cannabidiol (eCBD/vasel), hydrocortisone 1% (hydro/vasel), or vehicle–Vaseline (vasel), respectively, were applied from day 11. Treatment with an ointment containing eCBD resulted in a decrease in the number of leukocytes. The control group consisted of animals without inflammation. In the graphs, the bars filled in with gray correspond to groups sensitized with DNCB. Abbreviations: GRAN—granulocytes, GRA%—percentage of granulocytes, LYM—lymphocytes, LYM%—percentage of lymphocytes, MONO—monocytes, MONO%—percentage of monocytes, WBC—white blood cells, * *p* < 0.05.

Due to the above results, tests were also performed to examine changes in the percentages of individual populations of leukocytes. In the group with dermatitis treated with hydrocortisone, a significant increase in the percentage of monocytes (MON%) was observed relative to the negative control, but there was no effect on lymphocytes or granulocytes (Figure 7).

#### 2.4.2. Analysis of the Red Blood Cell Parameters

In the evaluation of red blood cell parameters, it was observed that the induction of dermatitis and treatment with Vaseline had no significant effect on the red blood cell system. Similarly, eCBD did not cause significant differences compared to either the healthy control or the Vaseline-treated group.

In turn, in rats with dermatitis treated with hydrocortisone, a significant increase in hematocrit (HCT) and hemoglobin (HGB) concentration was observed compared to both healthy rats and rats treated with Vaseline and eCBD. Therefore, it seems that this is a direct effect of hydrocortisone treatment and not an effect of atopic dermatitis.

Moreover, in rats treated with hydrocortisone ointment, an increase in the mean corpuscular volume of an erythrocyte (MCV) was observed compared to that in the group treated with Vaseline.

Furthermore, hydrocortisone treatment increased mean corpuscular hemoglobin (MCH) relative to the Vaseline, while the red blood cell count (RBC) in the hydrocortisone group was elevated only relative to the eCBD group.

**Figure 8:**
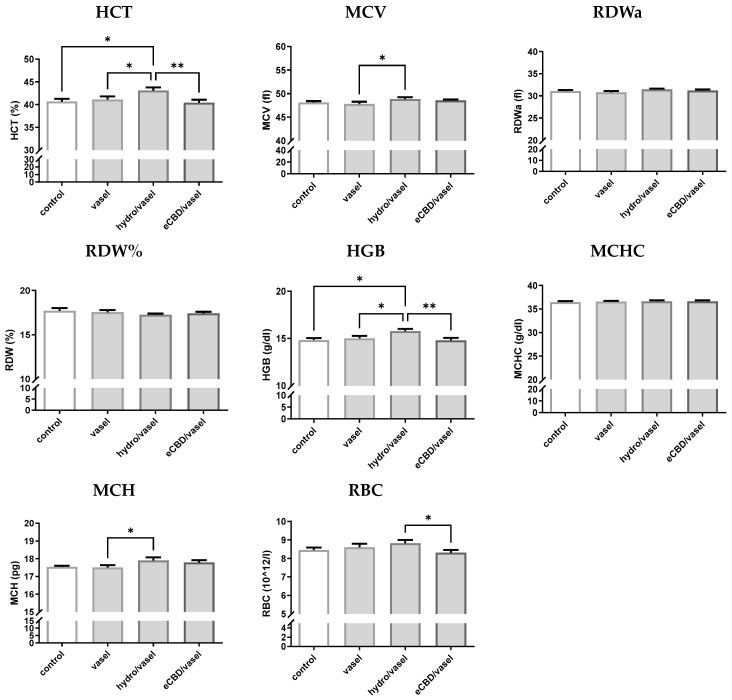
The effect of high-cannabidiol *Cannabis sativa* extract (eCBD) ointment on red blood cell parameters on DNCB-induced dermatitis. The 2,4-dinitrochlorobenzene (DNCB) solution was administered 9 times over a period of 3 weeks, and the tested ointments, which contained 2% cannabidiol (eCBD/vasel), hydrocortisone 1% (hydro/vasel), or vehicle–Vaseline (vasel), respectively, were applied from day 11. Changes in the red blood cell system were mainly observed in the hydrocortisone-treated group, and eCBD did not cause significant differences compared to control rats. The control group consisted of animals without inflammation. In the graphs, the bars filled in with gray correspond to groups sensitized with DNCB. Abbreviations: HCT—hematocrit, HGB—hemoglobin, MCH—mean corpuscular hemoglobin, MCHC—mean cell hemoglobin concentration, MCV—mean corpuscular volume of a red blood cell, RBC—red blood cell, RDWa—red cell distribution width—standard deviation, RDW%—red cell distribution width—coefficient of variation. * *p* < 0.05; ** *p* < 0.01.

Regarding the other parameters of the red blood cell system, i.e., the red cell distribution width–standard deviation (RDWa), red cell distribution width–coefficient of variation (RDW%), and mean cell hemoglobin concentration (MCHC), no significant differences between the groups were noted (Figure 8).

#### 2.4.3. Analysis of Platelet Parameters

**Figure 9:**
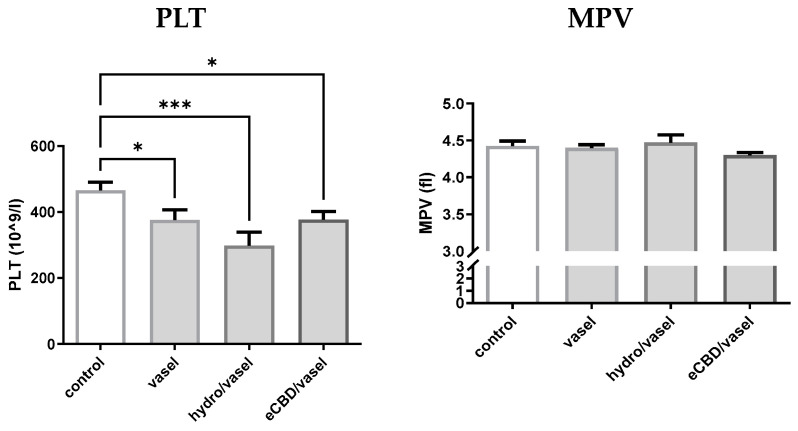
The effect of high-cannabidiol *Cannabis sativa* L. extract (eCBD) ointment on platelets parameters on DNCB-induced dermatitis. Rats were treated with eCBD ointment containing 2% cannabidiol (eCBD/vasel), Vaseline (vasel), and ointment containing 1% hydrocortisone (hydro/vasel), in 2,4-dinitrochlorobenzene (DNCB)-induced dermatitis model. The 2,4-dinitrochlorobenzene (DNCB) solution was administered 9 times over a period of 3 weeks, and the tested ointments, which contained 2% cannabidiol (eCBD/vasel), hydrocortisone 1% (hydro/vasel), or vehicle–Vaseline (vasel), respectively, were applied from day 11. It was observed that DNCB-induced dermatitis caused a significant decrease in the number of thrombocytes (PLT) in rats. However, no significant differences were observed between the groups treated with Vaseline, hydrocortisone, and eCBD. The control group consisted of animals without inflammation. In the graphs, the bars filled in with gray correspond to groups sensitized with DNCB. Abbreviations: MPV- mean platelet volume, PLT—platelet count, * *p* < 0.05; *** *p* < 0.001.

It was observed that DNCB-induced dermatitis caused a significant decrease in the number of thrombocytes (PLT) in rats. However, no significant differences were observed among the groups treated with Vaseline, hydrocortisone, and eCBD. In turn, the mean platelet volume (MPV) value did not change because of either the induction of atopic dermatitis or treatment with hydrocortisone or eCBD (Figure 9).

## 3. Discussion

In the present study, we observed that the ointment containing eCBD at a concentration corresponding to 2% CBD in the ointment effectively treated skin lesions in a rat model of dermatitis induced by topical application of DNCB. Interestingly, the anti-edematous effect of CBD occurred after only the first application of the compound and was clearly more intense than that in the group treated with hydrocortisone ointment. Furthermore, we confirmed for the first time that the anti-edematous effect of CBD in a rat model of dermatitis was accompanied by a reduction in the number of mast cells in the skin. Additionally, hematological analysis showed that local use of CBD had a significant effect on the white blood cell system.

Phytocannabinoids, especially CBD, are ingredients in numerous skin-care products recommended for applications such as eczema, acne, itching, and psoriasis, and numerous beneficial properties such as anti-inflammatory, moisturizing and even anti-wrinkle effects are attributed to them [23]. Dermatologists recommend the use of medical cannabis, most often in topical form, especially for patients with atopic dermatitis and psoriasis [24]. Unfortunately, despite the growing trend of using CBD for dermatological indications, scientific data are very limited, and so far the effectiveness of CBD has been evaluated in only a few preclinical studies [10,25,26,27,28,29] and in canine studies of dogs with atopic dermatitis [30,31,32], Dermatological indications have also been examined in human clinical studies [17,33,34,35,36,37,38]. However, the availability of research that compares CBD to other therapies commonly used in the clinic is very limited. In the present study, eCBD was compared to the glucocorticosteroid hydrocortisone, which allows for the assessment of both efficacy and potential adverse effects of eCBD compared to conventional AD therapy. Furthermore, in addition to observing local skin lesions, in the current study, we linked the effectiveness of eCBD in the treatment of dermatitis to analysis of blood parameters.

In our studies using a rat model of DNCB induced-dermatitis, we observed that eCBD ointment relieved ear edema significantly more effectively than did 1% hydrocortisone ointment. By contrast, Rundle et al. [27], in a mouse model of dermatitis induced by 12-O-Tetradecanoylphorbol-13-Acetate, observed that a topical formulation containing 0.9% cannabidiol and palmitoylethanolamide reduced ear edema to a lesser extent compared to the strong glucocorticosteroid mometasone. Glucocorticosteroids are currently, next to calcineurin inhibitors such as tacrolimus or pimecrolimus, the basis for the treatment of inflammatory lesions in atopic dermatitis. Unfortunately, the complex mechanism of action of glucocorticosteroids means that high anti-inflammatory effectiveness is accompanied by a number of serious adverse effects, both local (including thinning of the epidermis and dermis, hypopigmentation, atrophy of subcutaneous tissue, and telangiectasia) and systemic (such as hyperglycemia, dyslipidemia, osteoporosis, and cardiovascular disease). Interestingly, in our studies, local adverse reactions such as thinning of the skin, pallor of the integuments, and hair loss, which occurred in the hydrocortisone-treated group, were not observed in rats treated with eCBD. In conclusion, CBD, used in our studies at a concentration of 2%, showed higher anti-edematous effectiveness compared to hydrocortisone 1% and did not show the adverse effects typical of glucocorticoids, which suggests that the cannabinoid has a favorable therapeutic profile. However, a limitation of our study was the use of two different concentrations of the active substances, the cannabinoid and glucocorticoid.

The effect of reducing edema occurred after only the first application of eCBD ointment and was most pronounced in the first days of treatment with the ointment. Interestingly, despite a significant difference in ear thickness between rats treated with eCBD and rats treated with placebo (Vaseline), which persisted until the end of the experiment, no differences in the thickness of the epidermal layer were noted between these groups. In the course of AD, in the acute skin lesions, marked tissue edema (spongiosis) predominates. At this stage of AD, intercellular edema of epidermis is accompanied by intracellular edema, observed as keratinocytes ballooning [5]. In the chronic stage, hyperplastic epidermis and abnormal keratinocyte proliferation are observed as a result of processes such as *Staphylococcus aureus* skin colonization, recruitment of Th17 cells, and Il-17 cytokine production [39,40]. Therefore, it seems that eCBD does not have a significant effect on the remodeling of skin layers, although it is characterized by a pronounced anti-edematous effect.

eCBD, similarly to hydrocortisone, significantly reduced the number of mast cells in the affected skin, which confirms the effectiveness of the extract in alleviating allergic symptoms, since mast cells are a substantial component of the allergic reaction observed in dermatitis. Activated mast cells release histamine and other inflammatory mediators, resulting in vasodilation and increased vascular permeability, which manifest as skin redness and tissue edema. Furthermore, mast cells are located close to afferent neuron terminals, and mast cell-dependent mechanisms underlie the pruritus occurring in AD [41]. However, in our studies, eCBD, despite reducing the number of mast cells, did not significantly affect the frequency of scratching.

Pruritus is a very troublesome symptom in patients with atopic dermatitis. Therefore, research on substances that would effectively reduce the severity of itching is being conducted. Although the antipruritic effects of CBD have been suggested in the literature, only a few studies have confirmed the effectiveness of the cannabinoid in alleviating pruritus in humans and dogs with AD. Maghfour et al. [17] first reported the antipruritic effect of CBD after topical administration in AD patients. On the other hand, Mariga et al. [31] did not observe any differences in the degree of pruritus pre- and post-treatment in an evaluation of dogs with AD treated with full-spectrum high-CBD cannabis oil. Several studies have confirmed the reduction of itching symptoms in both humans and dogs with AD after the oral administration of cannabinoids as supplements [30,32,42]. Therefore, it cannot be ruled out that the reduction of itching in those cases was also influenced by the central effects of cannabinoids, such as the anxiolytic or sedative effects, which are characteristic of CBD. Loewinger et al. [30] observed that some dogs treated for AD that received mixed cannabidiol- and cannabidiolic acid-based oil (CBD/CBDA), administered p.o., experienced adverse events including lethargy and behavioral changes such as somnolence, sleepiness, decreased aggression, increased calmness, or increased energy/mobility.

Our results did not confirm a reduction in the frequency of scratching after administration of CBD, but, interestingly, we observed that the local application of eCBD slightly reduced the frequency of body shakes in rats. Transcutaneous permeation of CBD is very limited after topical application to the skin [38]. Therefore, it seems that the changes observed in the behavior of animals in our studies were not a direct effect of CBD on the central nervous system. However, the changes observed in the animals’ behavior, such as the reduced frequency of body shakes, could be due to the alleviation of the inflammatory process and the lower intensity of the perceived skin irritation, as was illustrated by the results of the histopathological analysis and blood analysis. On the other hand, damage to the epidermal barrier caused by scratching could potentially contribute to the increased absorption of the substance. Therefore, the changes in animal behavior observed in our study could have been partly the result of a systemic effects of CBD, e.g., reduced stress, as well of a reduction in the systemic inflammatory process.

A complete blood count revealed that local administration of eCBD caused changes in the values of parameters of the white blood cell system. It seems important that the profile of changes in the blood picture was different in the group treated with eCBD than in the hydrocortisone group, which suggests different mechanisms of anti-inflammatory action for the tested compounds.

The administration of eCBD caused a decrease in WBC values compared to the Vaseline group; this effect was not observed after hydrocortisone treatment. Moreover, a more detailed analysis of the white blood cell system showed a decrease in the number of lymphocytes in the eCBD group compared to the healthy control, which confirms the immunosuppressive effect of CBD described in the literature [43]. Ignatowska-Jankowska et al. [44] observed that systemic 14-day administration of CBD caused lymphopenia and a significant decrease in the number of T, B, T helper and T cytotoxic cells in Wistar rats. However, no decrease was observed in the NK and NKT lymphocyte subsets. On the other hand, Jani et al. [45], in a preliminary study performed in atopic and healthy dogs, observed only a minimal overall effect of CBD on the secretion of cytokines in peripheral blood mononuclear cells (PBMC). Cannabidiol modulates the functioning of the endocannabinoid system in a complex manner, and its impact on particular pathways is still not fully understood. The compound has low affinity for CB1 and CB2 receptors, but it has also been observed in some in vitro studies that it antagonizes the effects of agonists of these receptors [46,47]. Interestingly, studies conducted on knockout mice have shown that the immunomodulatory effect of CBD may be independent of cannabinoid receptors [48].

Phytocannabinoids can potentially modulate the inflammatory response by influencing receptors located on immune system cells, but also on other cells, such as keratinocytes or sensory nerves. Upregulation of cannabinoid CB2 receptors and cannabinoid-related receptors (TRPA1 and 5-HT1a) was observed in the skin epithelium in dogs with AD. Moreover, CB1, GPR55, TRPV1 and PPARα receptors were present in keratinocytes of healthy and AD dogs [21]. In turn, the presence of CB2, GPR55, TRPV1, and TRPA1 receptors was observed on immune system cells in the skin infiltrate of dogs with AD [22]. Given the fact that CBD interacts with these receptors and also alters the endogenous level of anandamide, the role of CBD in the potential regulation of physiological processes that change in the course of AD seems likely to be complex. However, further research is necessary. High CBD Cannabis sativa L. extract (eCBD) also tended to reduce the number of monocytes and granulocytes in the treated group compared to the AD group treated with Vaseline. Although this trend was statistically nonsignificant, it seems that CDB may have a beneficial effect on the treatment of AD due to the fact that macrophages and eosinophils are activated in chronic AD [5].

Treatment with eCBD, unlike treatment with hydrocortisone, did not show a significant effect on the parameters of the red blood cell system. The glucocorticosteroid significantly increased the hematocrit value compared not only to Vaseline (placebo), but also to the healthy control, and changed other parameters of the red blood cell system. This effect of glucocorticoids, probably related to the effect on erythropoiesis, is well documented and can be observed in, among others, patients with Cushing’s syndrome who are exposed to excess glucocorticoids. These patients have elevated values of hemoglobin, hematocrit, and red blood cell count [49]. In turn, analysis of platelet parameters revealed that neither eCBD nor hydrocortisone reversed the thrombocytopenia observed in all DNCB-induced dermatitis groups. Considering the different effects of eCBD and hydrocortisone on individual blood cell count parameters, the mechanism of the anti-inflammatory action of CBD seems to be different from that of a glucocorticosteroid.

The results of the present study indicate that eCBD has beneficial anti-edematous and anti-inflammatory effects; however, the model of DNCB-induced skin inflammation used has some limitations related to the pathogenesis of the disease. In human AD, the initial Th2 phase precedes the chronic phase, in which Th1 and Th0 lymphocytes predominate. The acute phase of the disease is associated with the production of cytokines by Th2 cells—interleukins 4, 5, and 13—and in the chronic phase, there is an increase in the levels of interferon gamma and interleukins 12 and 15 [6,50]. By contrast, repeated DNCB application in rats caused a shift of the initial Th1 response towards Th2, observed on days 7 and 21 of the experiment, respectively [51]. Moreover, another limitation in the study was the use of two different concentrations of active substances, 2% CBD and 1% hydrocortisone; therefore, it would be valuable to examine the anti-inflammatory potential of CBD depending on the concentration used. In addition, further studies are needed to assess the long-term efficacy of CBD in AD and the potential adverse effects of topically applied cannabinoid-containing extracts.

## 4. Materials and Methods

### 4.1. Animals

Experiments were performed on male Wistar rats (300–350 g). Animals were housed in cages lined with sawdust in a room maintained at a set temperature (22 ± 2 °C), humidity (55 ± 10%), and frequency of air changes (15–18 changes per 1 h) under a standard 12–12 h light/dark cycle, with free access to food and water. The animals were housed in groups of two to three in conventional cages. All animal procedures were approved by II Local Ethical Committee for Experiments on Animals at Warsaw University of Life Sciences (permit number: WAW2/120/2020) and performed in accordance with the guidelines published in Directive 2010/63/EU of the European Parliament and of the Council on the protection of animals used for scientific purposes. The total number of animals used in the study was 32 rats.

### 4.2. Drugs and Reagents

*Cannabis sativa* L. extract (Verdant Nature LLC branch in Warsaw, Poland), contained 26.6% CBD and <2% other cannabinoids (CBDV, CBG, 9-THC, CBC, CBE), was used to prepare the ointment containing 2% CBD and Vaseline (as a vehicle). The ointment containing 1% hydrocortisone (Pharma Cosmetic, Krakow, Poland) served as a positive control.

1-Chloro-2,4-dinitrobenzene (DNCB) was purchased from Sigma-Aldrich (St. Louis, MO, USA) and was dissolved in ethanol 70% to 1.5% (*w*/*v*) solution.

Hematoxylin (GHS332-1L) and eosin Y (HT110132-1L) were purchased from Sigma−Aldrich (St. Louis, MO, USA). Toluidine blue was purchased from Warchem (Warsaw, Poland).

### 4.3. Experimental Protocol

#### 4.3.1. Rat Model of Chronic Dermatitis

Dermatitis was induced by applying 1.5% DNCB solution to the right ear of the animal, based on the modified protocol described by Fuji et al. [51].

The choice of the rat model for the study is due to the great similarity of this species to humans in terms of skin structure and the course of inflammatory processes. The thickness of the stratum corneum of the epidermis, which affects, among other things, the permeability to applied substances, is very similar in rats and in humans. What is more, this similarity to humans is much greater for rats than for mice (15.4 µm in rats, 18.2 µm in humans, and 8.8 µm in mice [52].

**Scheme 1:**
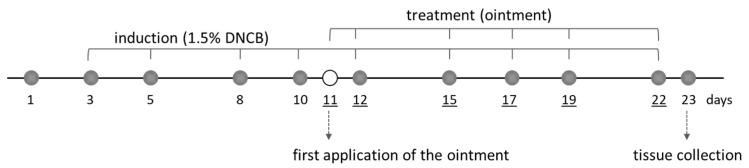
Experimental protocol for the induction of dermatitis by the application of 2,4-dinitrochlorobenzene (DNCB) and treatment with the *Cannabis sativa* L. extract (eCBD) ointment containing cannabidiol in rats. The 2,4-dinitrochlorobenzene (DNCB) solution was administered 9 times over a period of 3 weeks, and the tested ointments containing 2% cannabidiol (eCBD/vasel), hydrocortisone 1% (hydro/vasel), or vehicle–Vaseline (vasel) were applied beginning on day 11. The days on which the tested ointments were applied are underlined. The control group consisted of animals without inflammation.

In brief, the DNCB solution was applied in a volume of 60 µL to both sides of the animal’s right ear 9 times over a period of 3 weeks, on days 3, 5, 8, 10, 12, 15, 17, 19, and 22 (Scheme 1). In the negative control group, 70% ethanol was applied to the right ear of the animal.

Chronic dermatitis was characterized by the gradual development of skin inflammation, accompanied by edema, redness and significant pruritus. Irritation caused by topical administration of DNCB also manifested in symptoms like scratching, rubbing, excessive grooming or licking, and body shaking.

The severity of edema was assessed by measuring the ear thickness (mm) at the beginning of the experiment and on the selected days during the sensitization and treatment stages before DNCB application, using an electronic caliper. Intensity of the pruritus was assessed in behavioral observations for 10 min immediately after the application of DNCB on days 1, 3, 5, 8, 10, 12, 15, 17, 19, and 22 of the experiment. The number of scratching events, defined as one or more characteristic scratching movements with the paw directed toward the right ear of the animal, was counted. In addition, the body shakes of the animal were counted in the indicated time intervals.

#### 4.3.2. Preparation of the Ointment Containing High-Cannabidiol *Cannabis sativa* L. Extract

The ointment was prepared using vaselinum as a vehicle, with the addition of eCBD. For this purpose, eCBD was weighed in an amount that corresponded to 2%CBD content in the final product. The ointment ingredients were thoroughly mixed using a pharmaceutical mortar to obtain a homogeneous, uniform formulation. In order to maintain the sterility of the product, the ointment was divided into individual mini-products intended for application on individual days of the experiment.

#### 4.3.3. Application of the Ointment Containing High-Cannabidiol *C. sativa* L. Extract

The animals were randomly divided into four experimental groups: three groups with dermatitis, which was treated with Vaseline, eCBD ointment, or hydrocortisone ointment, as well as a negative control group (without dermatitis). Each experimental group consisted of seven to eight animals. *Cannabis sativa* L. ointment containing 2% CBD was applied after the initial period of sensitization, beginning on day 11, i.e., one day before the application of DNCB, on day 12. Then, the application of the ointment was repeated 6 h after each DNCB application, i.e., on days 11, 12, 15, 17, 19, and 22 (Scheme 1). The ointment was applied in a thin layer, in a total volume of 20 μL, to both sides of the right ear of the animals, using a microlaboratory sampling spoon. The therapeutic potential of eCBD ointment was assessed in comparison treatment with the vehicle Vaseline and 1% hydrocortisone ointment on the same schedule.

### 4.4. Histological Evaluation

The rats’ ears were collected one day after the end of behavioral experiments. The ear tissues were fixed in 10% neutral buffered formalin solution for 48 h (Sigma-Aldrich, USA) and then transferred to 70% ethanol. Tissues were then dehydrated in an ascending ethanol gradient, cleared in xylene, and embedded in paraffin. Sections of tissue were obtained on a Hyrax M25 microtome (Zeiss, Jena, Germany) with the slice thickness set to 5 µm.

Then, tissues were stained with hematoxylin-eosin (H&E) according to the standard protocol. Briefly, sections were deparaffinized in xylene, then rehydrated in descending ethanol gradient. In the next step, slices were stained in hematoxylin solution for 1.5 min, then in eosin for 15 s. Next, slides were dehydrated in a descending ethanol gradient, cleared in xylene, sealed with resinous mounting medium (DPX), and left under a fume hood to dry. Finally, all slides were photographed under final magnifications of 40× and 200×. In order to assess the effect of tested compounds on the inflammatory and skin-regeneration processes, the epidermal-layer thickness (µm) of the tissue was measured by two independent observers. The results are expressed as the mean of 5 measurements.

To detect mast cells, tissue sections were stained with toluidine blue according to the standard protocol. Briefly, sections were deparaffinized and dehydrated. Then, slides were stained in toluidine working solution (0.1% toluidine blue in 1% sodium chloride, pH 2.0–2.5). Finally, stained sections were quickly dehydrated, cleared in xylene, sealed with DPX, and left to dry. Then, slides were photographed under final magnifications of 40× and 200× and the mast cell number per mm^2^ was counted by two independent observers.

### 4.5. Blood Sample Analyses

Blood samples were collected one day after the end of the behavioral experiments and were collected directly from the hearts of anesthetized rats (isoflurane anesthesia) into BD Vacutainer ^®^ heparin tubes. Then, they were used to obtain complete blood counts on a Boule EXIGO veterinary hematology analyzer within 2 h of collection.

### 4.6. Statistical Analysis

The results are expressed as the mean ± standard error of the mean (SEM). In the behavioral studies, two-way analysis of variance (ANOVA) was used for statistical analysis. Comparisons between individual groups were performed using Fisher’s least significant difference (LSD) post-hoc test. Histopathological data and hematological parameters were analyzed using one-way analysis of variance (ANOVA), followed by Fisher’s least significant difference (LSD) post-hoc test. *p* values less than 0.05 were considered statistically significant. Analyses were performed using GraphPad Prism 9.

## 5. Conclusions

Taken together, the results of this study demonstrate that *Cannabis sativa* L. extract containing a high concentration of CBD (eCBD), applied topically in the form of an ointment, showed anti-inflammatory effects, as manifested in a reduction in ear edema in rats with DNCB-induced dermatitis. Interestingly, the anti-edematous effect of eCBD was more pronounced than that observed after hydrocortisone treatment at the concentrations of the substance used. Furthermore, eCBD caused a decrease in the number of mast cells in the inflamed skin and changes in the parameters of the white blood cell system. Therefore, it seems that eCBD may be a valuable addition to therapy in AD patients, but further research is needed.

## Data Availability

Data is included in this published article.

## Abbreviations

AD	atopic dermatitis
CBC	cannabichromene
CBD	cannabidiol
CBDV	cannabidivarin
CBE	cannabielsoin
CBG	cannabigerol
DNCB	2,4-dinitrochlorobenzene
eCBD	cannabidiol-enriched *Cannabis sativa* L. extract
GRA%	percentage of granulocytes
GRAN	granulocytes
HCT	hematocrit
HGB	hemoglobin
IgE	immunoglobulin E
LYM	lymphocytes
LYM%	percentage of lymphocytes
MCH	mean corpuscular hemoglobin
MCHC	mean cell hemoglobin concentration
MCV	mean corpuscular volume of a red blood cell
MONO	monocytes
MONO%	percentage of monocytes
MPV	mean platelet volume
PBMC	peripheral blood mononuclear cells
PEA	palmitoylethanolamide
PLT	platelet count
RBC	red blood cell
RDW%	red cell distribution width–coefficient of variation
RDWa	red cell distribution width–standard deviation
THC	Δ-9-tetrahydrocannabinol
WBC	white blood cells

## References

[1] Novak N., Bieber T., Leung D.Y. (2003). Immune mechanisms leading to atopic dermatitis. J. Allergy Clin. Immunol..

[2] Blome C., Radtke M.A., Eissing L., Augustin M. (2016). Quality of Life in Patients with Atopic Dermatitis: Disease Burden, Measurement, and Treatment Benefit. Am. J. Clin. Dermatol..

[3] David Boothe W., Tarbox J.A., Tarbox M.B. (2017). Atopic Dermatitis: Pathophysiology. Adv. Exp. Med. Biol..

[4] Eckert E., Ruzicka T., Ring J., Przybilla B. (1991). Histopathological and Immunohistological Aspects of Atopic Eczema. Handbook of Atopic Eczema.

[5] Leung D.Y. (1995). Atopic dermatitis: The skin as a window into the pathogenesis of chronic allergic diseases. J. Allergy Clin. Immunol..

[6] Bieber T. (2010). Atopic dermatitis. Ann. Dermatol..

[7] Buys L.M. (2007). Treatment options for atopic dermatitis. Am. Fam. Physician.

[8] Maurer M., Worm M., Zuberbier T., Reitamo S., Luger T.A., Steinhoff M. (2008). Antihistamines in atopic dermatitis. Textbook of Atopic Dermatitis.

[9] Wilkinson J.D., Williamson E.M. (2007). Cannabinoids inhibit human keratinocyte proliferation through a non-CB1/CB2 mechanism and have a potential therapeutic value in the treatment of psoriasis. J. Dermatol. Sci..

[10] Tubaro A., Giangaspero A., Sosa S., Negri R., Grassi G., Casano S., Della Loggia R., Appendino G. (2010). Comparative topical anti-inflammatory activity of cannabinoids and cannabivarins. Fitoterapia.

[11] Gaffal E., Cron M., Glodde N., Tüting T. (2013). Anti-inflammatory activity of topical THC in DNFB-mediated mouse allergic contact dermatitis independent of CB1 and CB2 receptors. Allergy.

[12] Ligresti A., De Petrocellis L., Di Marzo V. (2016). From Phytocannabinoids to Cannabinoid Receptors and Endocannabinoids: Pleiotropic Physiological and Pathological Roles Through Complex Pharmacology. Physiol. Rev..

[13] Martinez Naya N., Kelly J., Corna G., Golino M., Abbate A., Toldo S. (2023). Molecular and Cellular Mechanisms of Action of Cannabidiol. Molecules.

[14] Marinho A., da Silva-Neto R. (2023). Anti-inflammatory effects of cannabinoids. Braz. J. Pain.

[15] Martinelli G., Magnavacca A., Fumagalli M., Dell’Agli M., Piazza S., Sangiovanni E. (2022). *Cannabis sativa* and Skin Health: Dissecting the Role of Phytocannabinoids. Planta Med..

[16] Del Rosso J.Q. (2007). Use of a palmitoylethanolamide-containing nonsteroidal cream for treating atopic dermatitis: Impact on the duration of response and time between flares. Cosmet. Dermatol..

[17] Maghfour J., Rundle C.W., Rietcheck H.R., Dercon S., Lio P., Mamo A., Runion T.M., Fernandez J., Kahn J., Dellavalle R.P. (2021). Assessing the Effects of Topical Cannabidiol in Patients with Atopic Dermatitis. Dermatol. Online J..

[18] Campora L., Miragliotta V., Ricci E., Cristino L., Di Marzo V., Albanese F., Federica Della Valle M., Abramo F. (2012). Cannabinoid receptor type 1 and 2 expression in the skin of healthy dogs and dogs with atopic dermatitis. Am. J. Vet. Res..

[19] Abramo F., Campora L., Albanese F., della Valle M.F., Cristino L., Petrosino S., Di Marzo V., Miragliotta V. (2014). Increased levels of palmitoylethanolamide and other bioactive lipid mediators and enhanced local mast cell proliferation in canine atopic dermatitis. BMC Vet. Res..

[20] Martín-Fontecha M., Eiwegger T., Jartti T., Rueda-Zubiaurre A., Tiringer K., Stepanow J., Puhakka T., Rückert B., Ortega-Gutiérrez S., López-Rodríguez M.L. (2014). The expression of cannabinoid receptor 1 is significantly increased in atopic patients. J. Allergy Clin. Immunol..

[21] Chiocchetti R., De Silva M., Aspidi F., Cunha R.Z., Gobbo F., Tagliavia C., Sarli G., Morini M. (2022). Distribution of Cannabinoid Receptors in Keratinocytes of Healthy Dogs and Dogs with Atopic Dermatitis. Front. Vet. Sci..

[22] Chiocchetti R., Salamanca G., De Silva M., Gobbo F., Aspidi F., Cunha R.Z., Galiazzo G., Tagliavia C., Sarli G., Morini M. (2022). Cannabinoid receptors in the inflammatory cells of canine atopic dermatitis. Front. Vet. Sci..

[23] Jhawar N., Schoenberg E., Wang J.V., Saedi N. (2019). The growing trend of cannabidiol in skincare products. Clin. Dermatol..

[24] Yeroushalmi S., Nelson K., Sparks A., Friedman A. (2020). Perceptions and recommendation behaviors of dermatologists for medical cannabis: A pilot survey. Complement. Ther. Med..

[25] Petrosino S., Verde R., Vaia M., Allarà M., Iuvone T., Di Marzo V. (2018). Anti-inflammatory Properties of Cannabidiol, a Nonpsychotropic Cannabinoid, in Experimental Allergic Contact Dermatitis. J. Pharmacol. Exp. Ther..

[26] Casares L., García V., Garrido-Rodríguez M., Millán E., Collado J.A., García-Martín A., Peñarando J., Calzado M.A., de la Vega L., Muñoz E. (2020). Cannabidiol induces antioxidant pathways in keratinocytes by targeting BACH1. Redox Biol..

[27] Rundle C.W., Rietcheck H.R., Maghfour J., Dercon S., Fernandez J., Lio P., Dellavalle R.P., Fujita M., Yardley H. (2022). Anti-inflammatory Effect of Cannabidiol and Palmitoylethanolamide Containing Topical Formulation on Skin in a 12-O-Tetradecanoylphorbol-13-Acetate-Induced Dermatitis Model in Mice. Dermatitis.

[28] Sangiovanni E., Fumagalli M., Pacchetti B., Piazza S., Magnavacca A., Khalilpour S., Melzi G., Martinelli G., Dell’Agli M. (2019). *Cannabis sativa* L. extract and cannabidiol inhibit in vitro mediators of skin inflammation and wound injury. Phytother. Res..

[29] Massimini M., Dalle Vedove E., Bachetti B., Di Pierro F., Ribecco C., D’Addario C., Pucci M. (2021). Polyphenols and Cannabidiol Modulate Transcriptional Regulation of Th1/Th2 Inflammatory Genes Related to Canine Atopic Dermatitis. Front. Vet. Sci..

[30] Loewinger M., Wakshlag J.J., Bowden D., Peters-Kennedy J., Rosenberg A. (2022). The effect of a mixed cannabidiol and cannabidiolic acid based oil on client-owned dogs with atopic dermatitis. Vet. Dermatol..

[31] Mariga C., Souza Silva Mateus A.L., Dos Santos Dullius Â.I., da Silva A.P., Martins Flores M., Vasconcelos Soares A., Amazonas E., Tadeu Lemos Pinto Filho S. (2023). Dermatological evaluation in dogs with atopic dermatitis treated with full-spectrum high cannabidiol oil: A pre study part 1. Front. Vet. Sci..

[32] Mogi C., Yoshida M., Kawano K., Fukuyama T., Arai T. (2022). Effects of Cannabidiol Without Delta-9-Tetrahydrocannabinol on Canine Atopic Dermatitis: A Retrospective Assessment of 8 Cases. Can. Vet. J..

[33] Maghfour J., Rietcheck H.R., Rundle C.W., Runion T.M., Jafri Z.A., Dercon S., Lio P., Fernandez J., Fujita M., Dellavalle R.P. (2020). An Observational Study of the Application of a Topical Cannabinoid Gel on Sensitive Dry Skin. J. Drugs Dermatol..

[34] Maghfour J., Rietcheck H., Szeto M.D., Rundle C.W., Sivesind T.E., Dellavalle R.P., Lio P., Dunnick C.A., Fernandez J., Yardley H. (2021). Tolerability profile of topical cannabidiol and palmitoylethanolamide: A compilation of single-centre randomized evaluator-blinded clinical and in vitro studies in normal skin. Clin. Exp. Dermatol..

[35] Palmieri B., Laurino C., Vadala M. (2019). A therapeutic effect of cbd-enriched ointment in inflammatory skin diseases and cutaneous scars. Clin. Ter..

[36] Gao Y., Li Y., Tan Y., Liu W., Ouaddi S., McCoy J., Kovacevic M., Situm M., Stanimirovic A., Li M. (2022). Novel cannabidiol aspartame combination treatment (JW-100) significantly reduces ISGA score in atopic dermatitis: Results from a randomized double-blinded placebo-controlled interventional study. J. Cosmet. Dermatol..

[37] Kuzumi A., Yamashita T., Fukasawa T., Yoshizaki-Ogawa A., Sato S., Yoshizaki A. (2024). Cannabinoids for the treatment of autoimmune and inflammatory skin diseases: A systematic review. Exp. Dermatol..

[38] Kuzumi A., Yoshizaki-Ogawa A., Fukasawa T., Sato S., Yoshizaki A. (2024). The Potential Role of Cannabidiol in Cosmetic Dermatology: A Literature Review. Am. J. Clin. Dermatol..

[39] Kim J., Kim B.E., Leung D.Y.M. (2019). Pathophysiology of atopic dermatitis: Clinical implications. Allergy Asthma Proc..

[40] Kader H.A., Azeem M., Jwayed S.A., Al-Shehhi A., Tabassum A., Ayoub M.A., Hetta H.F., Waheed Y., Iratni R., Al-Dhaheri A. (2021). Current Insights into Immunology and Novel Therapeutics of Atopic Dermatitis. Cells.

[41] Kawakami T., Ando T., Kimura M., Wilson B.S., Kawakami Y. (2009). Mast cells in atopic dermatitis. Curr. Opin. Immunol..

[42] Callaway J., Schwab U., Harvima I., Halonen P., Mykkänen O., Hyvönen P., Järvinen T. (2005). Efficacy of dietary hempseed oil in patients with atopic dermatitis. J. Dermatolog Treat..

[43] Peyravian N., Deo S., Daunert S., Jimenez J.J. (2020). Cannabidiol as a Novel Therapeutic for Immune Modulation. Immunotargets Ther..

[44] Ignatowska-Jankowska B., Jankowski M., Glac W., Swiergel A.H. (2009). Cannabidiol-induced lymphopenia does not involve NKT and NK cells. J. Physiol. Pharmacol..

[45] Jani T., Santoro D., Shmalberg J. (2025). Investigation of the in vitro effects of cannabidiol, cannabidiolic acid, and the terpene β-caryophyllene on lymphocytes harvested from atopic and healthy dogs: A preliminary study. Res. Vet. Sci..

[46] Thomas A., Baillie G.L., Phillips A.M., Razdan R.K., Ross R.A., Pertwee R.G. (2007). Cannabidiol displays unexpectedly high potency as an antagonist of CB1 and CB2 receptor agonists in vitro. Br. J. Pharmacol..

[47] Pertwee R.G. (2008). The diverse CB1 and CB2 receptor pharmacology of three plant cannabinoids: Δ9-tetrahydrocannabinol, cannabidiol and Δ9-tetrahydrocannabivarin. Br. J. Pharmacol..

[48] Kaplan B.L., Springs A.E., Kaminski N.E. (2008). The profile of immune modulation by cannabidiol (CBD) involves deregulation of nuclear factor of activated T cells (NFAT). Biochem. Pharmacol..

[49] Miller W.L., Tyrrell J.B., Fehlig P., Baxter J.D., Frohman L.A. (1995). The Adrenal Cortex. Endocrinology and Metabolism.

[50] Riedl R., Kühn A., Rietz D., Hebecker B., Glowalla K.G., Peltner L.K., Jordan P.M., Werz O., Lorkowski S., Wiegand C. (2023). Establishment and Characterization of Mild Atopic Dermatitis in the DNCB-Induced Mouse Model. Int. J. Mol. Sci..

[51] Fujii Y., Takeuchi H., Sakuma S., Sengoku T., Takakura S. (2009). Characterization of a 2,4-dinitrochlorobenzene-induced chronic dermatitis model in rats. Skin. Pharmacol. Physiol..

[52] Sato K., Sugibayashi K., Morimoto Y. (1991). Species differences in percutaneous absorption of nicorandil. J. Pharm. Sci..

